# Supplementary material for: Extending quantum-mechanical benchmark accuracy to biological ligand-pocket interactions
Source: Nat Commun. 2025 Sep 29;16:8583. doi: 10.1038/s41467-025-63587-9 (PMC12479982; doi:10.1038/s41467-025-63587-9)
Supplement: Supplementary file 1 — Supplementary Information [file 41467_2025_63587_MOESM1_ESM.pdf]

Supplementary Information: Extending quantum-mechanical benchmark accuracy to biological ligand-pocket interactions

Mirela Puleva[1, 2], Leonardo Medrano Sandonas[3,*], Balázs D. Lőrincz[4,5,6], Jorge Charry[1, 7], David M. Rogers[8], Péter R. Nagy[4,5,6, *], and Alexandre Tkatchenko[1, 2, *]

[1]Department of Physics and Materials Science, University of Luxembourg, L-1511 Luxembourg, Luxembourg.
[2]Institute for Advanced Studies, University of Luxembourg, Campus Belval, L-4365 Esch-sur-Alzette, Luxembourg.
[3]Institute for Materials Science and Max Bergmann Center of Biomaterials, TUD Dresden University of Technology, 01062 Dresden, Germany.
[4]Department of Physical, Chemistry and Materials Science, Faculty of Chemical Technology and Biotechnology, Budapest University of Technology and Economics, H-1111 Budapest, Hungary
[5]HUN-REN-BME Quantum Chemistry Research Group, H-1111 Budapest, Hungary
[6]MTA-BME Lendület Quantum Chemistry Research Group, H-1111 Budapest, Hungary
[7]Luxembourg Researchers Hub asbl, 223 rue de Luxembourg, L-4222, Esch-sur-Alzette, Luxembourg
[8]National Center for Computational Sciences, Oak Ridge National Laboratory, Oak Ridge, Tennessee, USA
[*]Corresponding authors: Leonardo Medrano Sandonas (leonardo.medrano@tu-dresden.de), Péter R. Nagy (nagy.peter@vbk.bme.hu), and Alexandre Tkatchenko (alexandre.tkatchenko@uni.lu).

1 QUID description and analysis

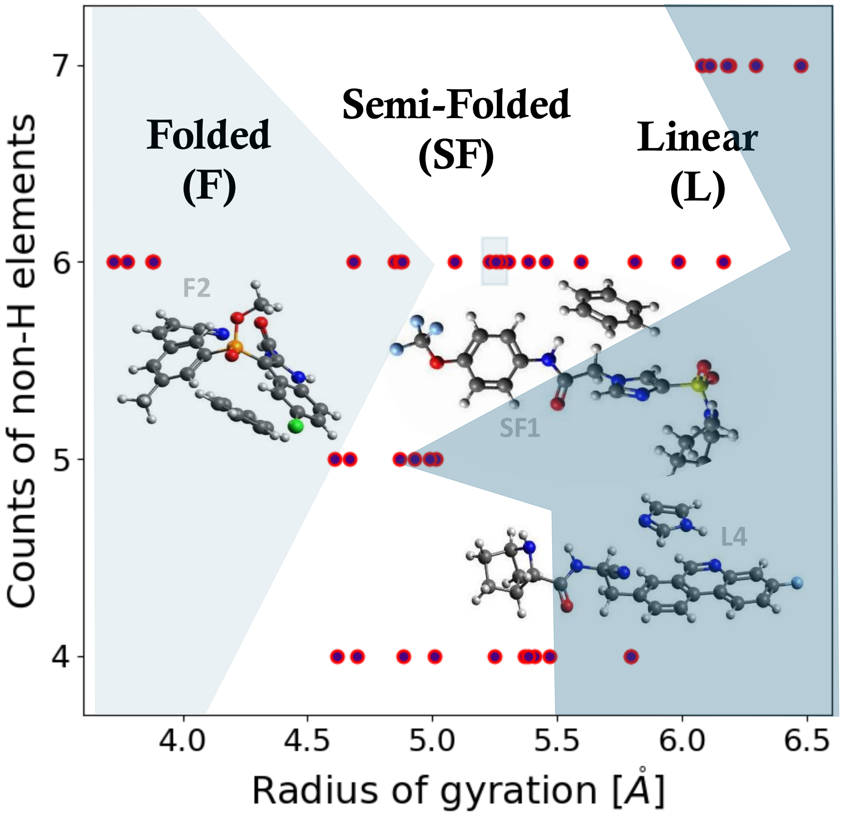

Figure S1. The number of different heavy (non-Hydrogen) elements in a dimer versus the radius of gyration for all 42 equilibrium QUantum Interacting Dimer (QUID) structures. The 'Folded', 'Semi-folded', and 'Linear' geometry categories are shown on the scatter plot as well.

The biggest difference between LNO-CCSD(T)/CBS and sSAPT0/jaDZ is found for the F1I1 dimer at 1.97 kcal/mol, with a scatter plot with comparison of the results for all equilibrium dimers presented in Fig. S3 of the SI. The difference between LNO-CCSD(T)/CBS and sSAPT0/jaDZ is within the uncertainty estimate of the LNO-CCSD(T)/CBS method for 7 dimers: L3B3, L3I3, L1B2, L1I2, L2I3, SF3I1, and F1I3 as shown on Fig. S3 of the SI. For 15 dimers the difference is over 1 kcal/mol, of which for 10 dimers within 1.5 kcal/mol: F2B1 (1.3), F2B2 (1.2), L3B1 (1.2), L3B2 (1.3), L1I1 (1.1), L2B1 (1.2), L2B3 (1.3), F1I2(1.5), and L4B1 (1.1), while for 6 dimers the difference is between 1.5 kcal/mol and 2 kcal/mol: F2I1 (1.7), F2I2 (1.6), SF1I2 (1.6), SF2I3 (1.9), F1I1 (2.0), and L4I1 (1.7).

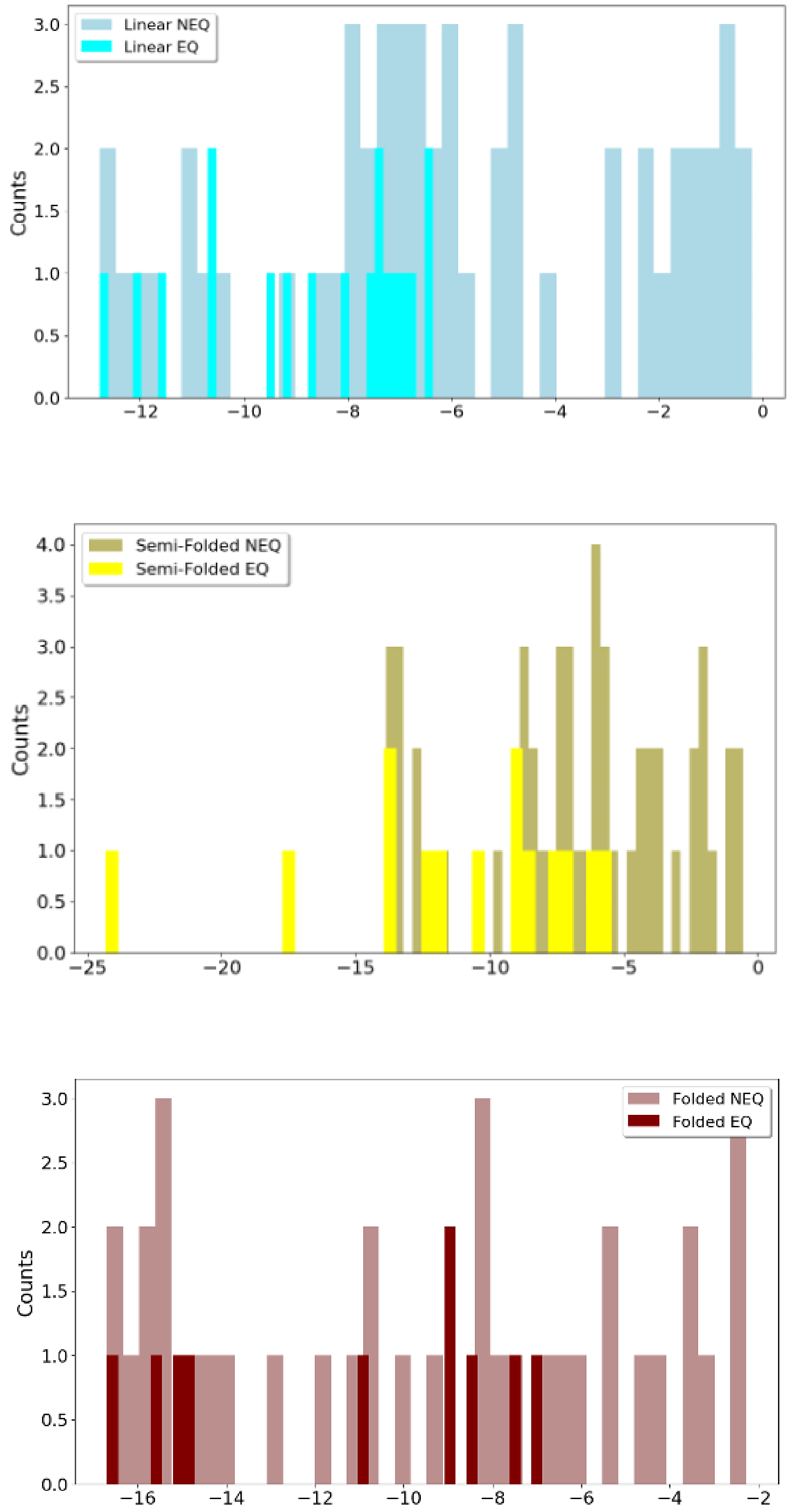

Figure S2. QUantum Interacting Dimer (QUID) results for the interaction energy at PBE0+Many-Body Dispersion by category–from top to bottom Linear, Semi-Folded, and Folded for 42 equilibrium and all 128 non-equilibrium configurations.

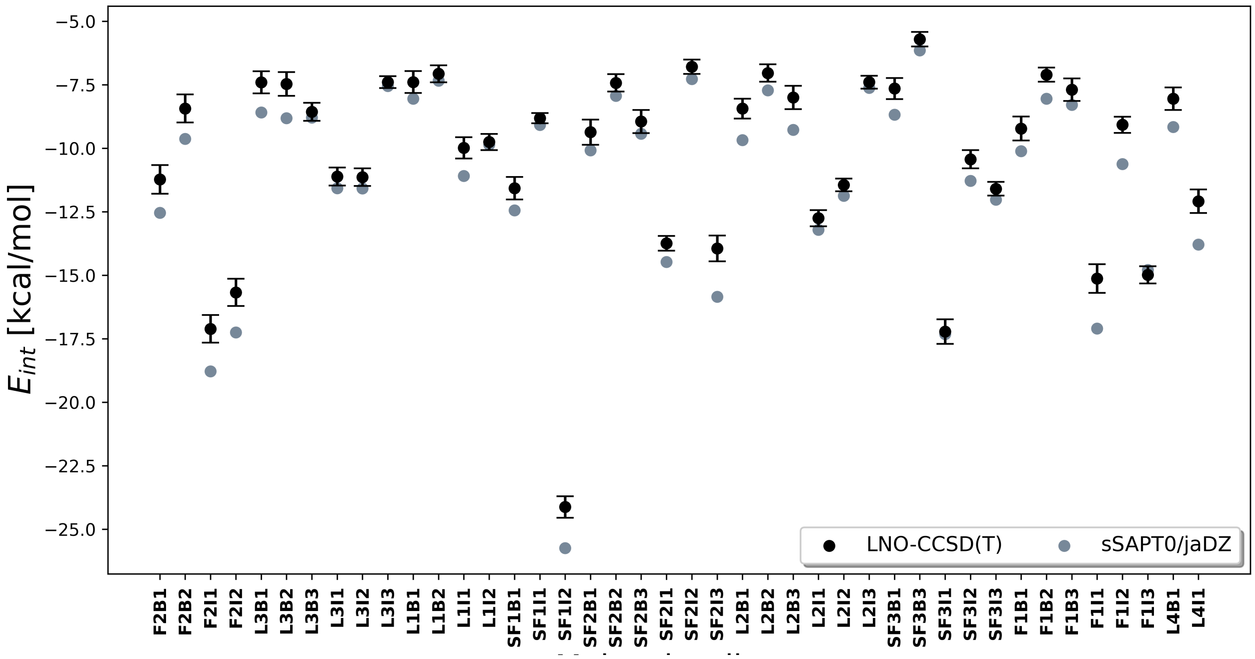

Figure S3. QUantum Interacting Dimer (QUID) structures results for the interaction energy at the Symmetry-adapterd perturbation theory level, sSAPT0/jaDZ, compared to a Local Natural Orbitals - Coupled Cluster with Singles, Doubles, and perturbative triplets (LNO-CCSD(T)) reference (provided with uncertainty estimate.

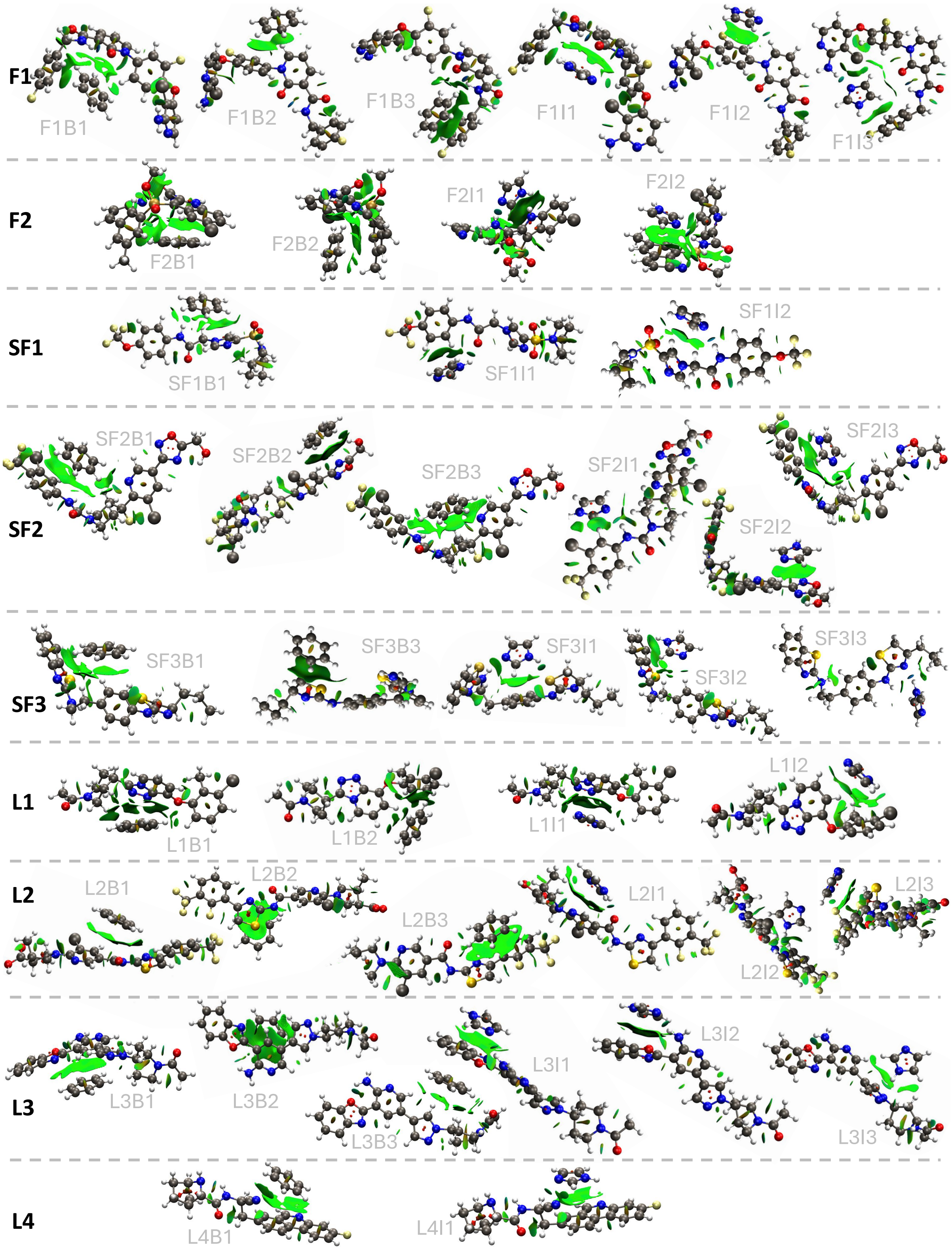

Figure S4. QUantum Interacting Dimer (QUID) dataset: analysis of non-covalent bonds in all equilibrium dimers with NCI-plot software.

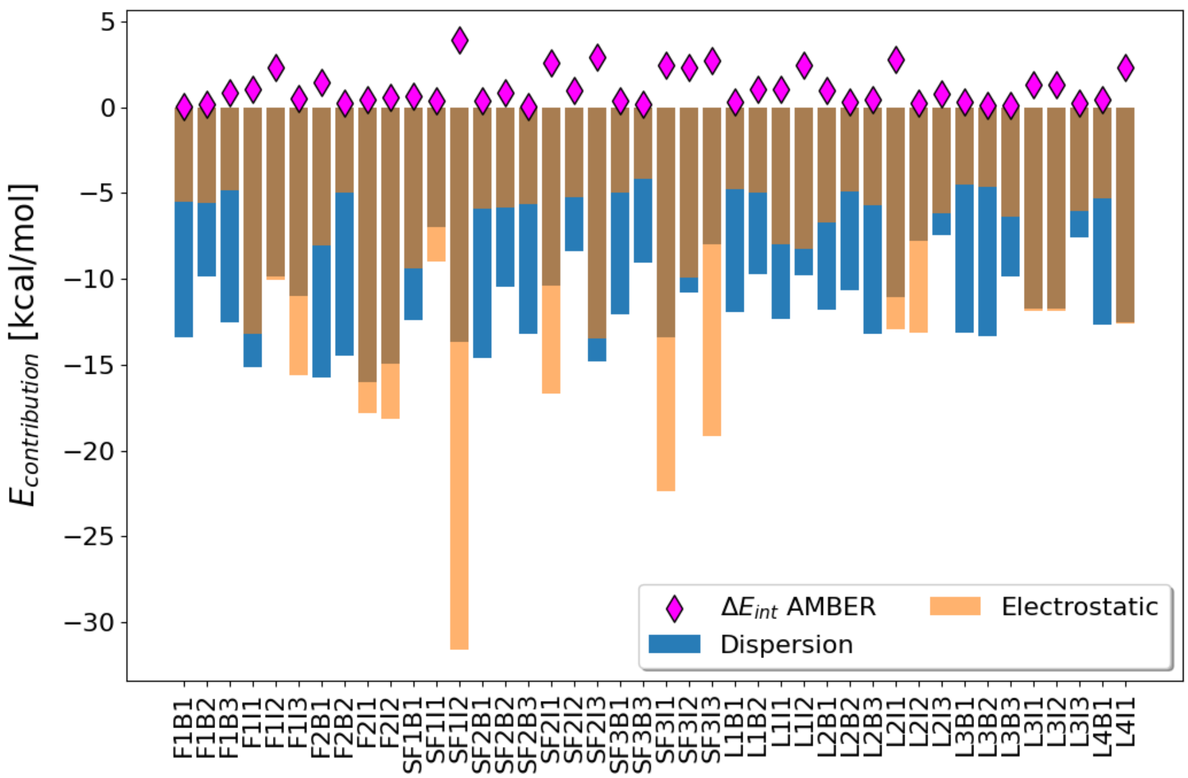

Figure S5. QUantum Interacting Dimer (QUID) molecular dimers dataset analysis of non-covalent bonds in all equilibrium dimers with Symmetry-adapted perturbation theory sSAPT0 method implemented in Psi4 for all equilibrium dimers. QUID molecular dimers dataset analysis of non-covalent bonds in all equilibrium dimers with sSAPT0 method compared to the AMBER-GAFF2 results with the interaction energies w.r.t results from Local Natural Orbitals - Coupled Cluster with Singles, Doubles, and perturbative triplets (LNO-CCSD(T)).

2 QUID Interaction energy predictions

For 6 dimers the interaction is underestimated for PBE0+MBD (L3B2, L1I1, SF2I2, L2B2, L2B3, and L4B1), while it is is overestimated for 8 others by PBE0+D4 (F2I2, SF1B1, SF1I2, L2I1, SF3I1, SF3I2, F1I3, L4I1).

The MAE results for the compressed and elongated regimes were calculated by averaging over the absolute of the differences to avoid misleading results due to summing up positive and negative errors (i.e. over- and under-binding). While both PBE0+MBD and PBEO+D4 display good results (slightly lower MAE for PBE0+D4), a case-by-case investigation can give us a more informative look on their specific predictions and errors. Among the exceptions, which lie outside of the error bars for shorter inter-monomer distances, are the predictions for PBE0+MBD (especially at q=0.9 and q=0.95), while larger distances are found more challenging for the PBEO+D4 method (especially at q=1.10 and q=1.25). Notably, for both methods at the largest distance of q=2.0, i.e. between 5-7 Å, all dimers are captured correctly within LNO-CCSD(T) uncertainty estimates except for SF2B2. Only F2I1 at q=0.9 distance for PBEO+MBD and F2B1 at q=1.25 for PBEO+D4 do not produce results within the predictions from LNO-CCSD(T) with the deviation being small at a fraction of a kcal/mol and not affecting the correct capture of the shape or the position of the minimum for the dissociation curves as seen on Fig. **??** and Fig. S9 - S14.

Interaction energy predictions outside of LNO-CCSD(T) error bars as seen on Fig. S9 - S14: at q=0.9 none for D4 and F2I1, L2B3, L2I3, SF2B2, and SF2I2 for MBD at q=0.95 none for D4 and L2B3, L2I3, SF2B2, and SF2I2 for MBD at q=1.00: SF2B2 for D4 and L2B3, SF2I2 for MBD at q=1.05: L2B3, L2I3, SF2I2 for D4, SF2B2, and SF2I2 for MBD at q=1.10: L2B3, L2I3, SF2B2, SF2I2 for D4 and L2B3, SF2I2 for MBD at q=1.25: F2B1, L2B3, L2I3, SF2B2, SF2I2 for D4 and none for MBD at q = 2.0: SF2B2 for both D4 and MBD

ab initio	MA(ΔE)[kcal/mol]	
LNO-CCSD(T)	0.39	-
DFT functional + dispersion	MAE [kcal/mol]	RMSE [kcal/mol]
GGA functionals		
PBE+MBD	0.44	0.51
Global hybrids		
PBE0+MBD	0.33	0.41
PBE0+D4	0.37	0.45
PBE0+XDM	0.56	0.60
M06-2X	0.62	0.73
PBE0+MBD-NL	0.72	0.81
B3LYP-D3	1.06	1.20
PBE0+TS	1.10	1.18
BH&HLYP+XDM	1.34	1.98
Range-separated hybrids		
ωB97X-V	0.35	0.41
ωB97M-V	0.53	0.63
ωB97X+D3	0.50	0.62
CAM-B3LYP+XDM	1.02	1.34
Double hybrids		
PBE-QIDH+D3	0.59	0.63
Semiempirical methods		
DFTB3+MBD	1.90	2.43
GFN2-xTB	0.89	1.13

Table S1. Mean Absolute Error (MAE) and Root Mean Square Error (RMSE) values for a variety of Density Functional Theory (DFT) and semiempirical methods w.r.t Local Natural Orbitals - Coupled Cluster with Singles, Doubles, and perturbative triplets (LNO-CCSD(T)) in kcal/mol for the 42 equilibrium QUantum Interacting Dimer (QUID) dimers, where the DFT functionals are listed with the added dispersion method, and ordered based on their type, and per type in ascending order of MAE values for the equilibrium QUID dimers
. The mean absolute value of the uncertainty estimate for the LNO-CCSD(T) results is also provided as a reference at the top.

Method	MA($\Delta E_{compressed}$)	MA($\Delta E_{elongated}$)
LNO-CCSD(T)	0.45	0.26
DFT functional + dispersion	MAE $E_{compressed}$	MAE $E_{elongated}$
GGA functionals		
PBE+MBD	0.84	0.20
Global hybrids		
PBE0+MBD	0.61	0.17
PBE0+D4	0.20	0.24
PBE0+XDM	0.93	0.23
PBE0+TS	0.83	0.59
PBE0+MBD-NL	1.10	0.44
M06-2X	2.12	0.60
Range-separated hybrids		
ωB97X-V	0.24	0.28
ωB97X+D3	0.35	0.37
ωB97M-V	0.70	0.27
Double hybrids		
PBE-QIDH+D3	1.13	0.41
Semiempirical and classical FF methods		
AMBER-GAFF2	1.13	0.41
GFN2-xTB	1.01	0.63
DFTB3+MBD	1.26	1.14

Table S2. Interaction energy MAE in kcal/mol for non-equilibrium dimers w.r.t. Local Natural Orbitals - Coupled Cluster with Singles, Doubles, and perturbative triplets (LNO-CCSD(T)) for a variety of methods: Density Functional Theory (DFT), semiempirical, and classical force fields, where the DFT functionals are listed with the added dispersion method, and ordered based on their type

. The non-equilibrium dimers are considered in two regimes: *compressed*, for which the non-covalent bond length is smaller or equal to the equilibrium one, and *elongated*, for which the non-covalent bond length is longer than in equilibrium. As a reference, also the mean of the LNO-CCSD(T) uncertainty estimates in the two regimes are provided at the top.

Dimer	Δ[kcal/mol]						
	PBE0+MBD	PBE0+D4	ωB97X-V	PBE0+XDM	AMBER-GAFF2	GFN2-xTB	DFTB3+MBD
F2B1	0.11	0.23	0.33	0.32	0.55	0.96	0.86
F2I1	0.23	0.20	0.33	0.44	0.36	1.67	2.59
SF2B2	0.16	0.21	0.21	0.20	0.39	0.36	0.92
SF2I2	0.23	0.22	0.24	0.23	0.31	0.36	0.98
L2B3	0.28	0.22	0.23	0.29	0.35	0.45	0.52
L2I3	0.10	0.22	0.16	0.14	0.70	0.10	0.48

Table S3. Delta metic (Δ) results for the dissociation curves w.r.t Local Natural Orbitals - Coupled Cluster with Singles, Doubles, and perturbative triplets (LNO-CCSD(T)) of the selection of 6 dimers F2B1, F2I1, SF2B2, SF2I2, L2B3, and L2I3 for the best Density Functional Theory (DFT), semiempirical, and classical force field methods investigated.

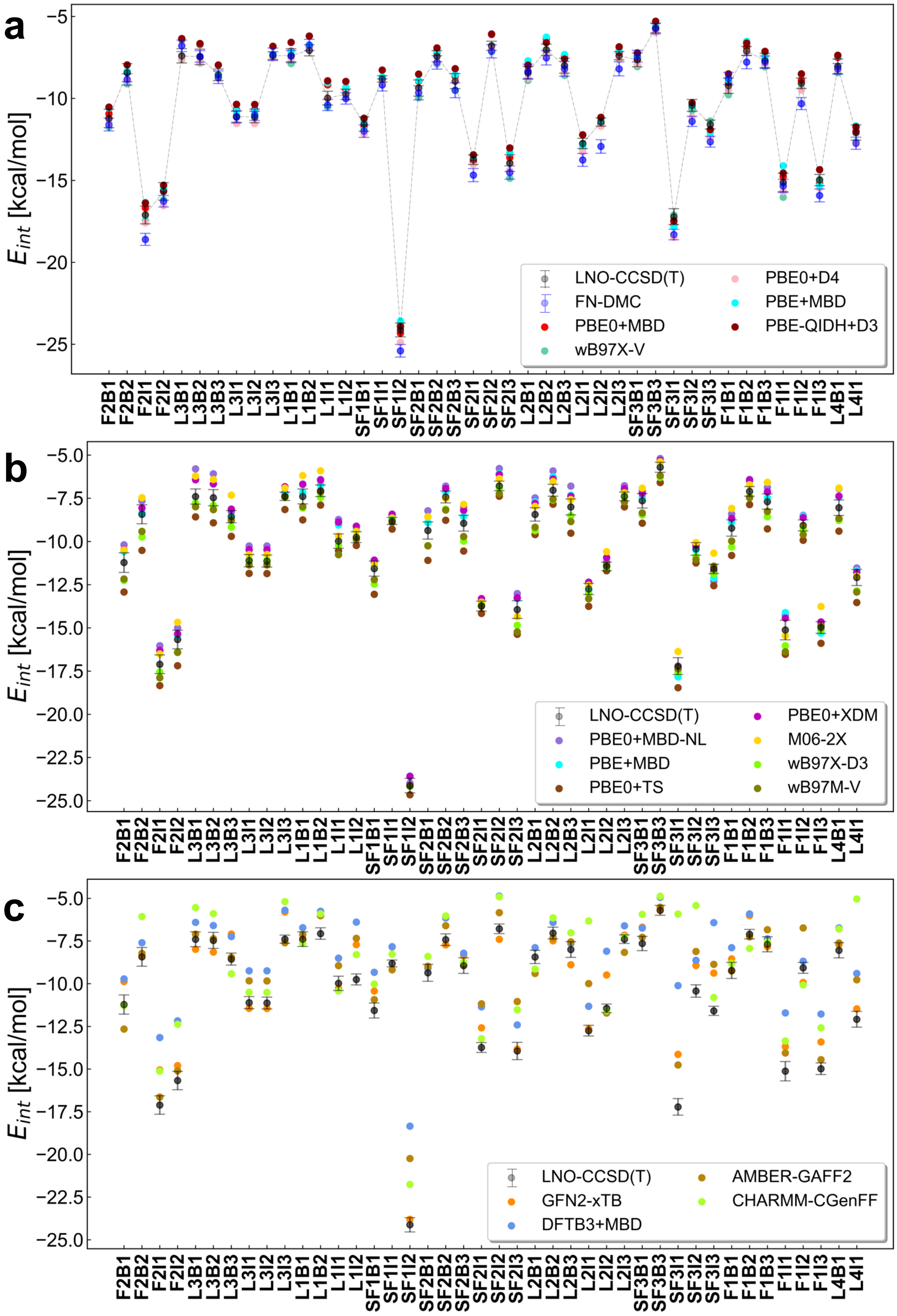

Figure S6. QUantum Interacting Dimer (QUID) 42 equilibrium structures: interaction energy E_{int} calculated with Local Natural Orbitals - Coupled Cluster with Singles, Doubles, and perturbative triplets (LNO-CCSD(T)) as reference and Fixed-node Diffusion Monte Carlo (FN-DMC)- both with uncertainty estimates, and the methods a) at Density Functional Theory, PBE0+Many-Body Dispersion (MBD), PBE0+D4, ωB97X-V, PBE+MBD, and PBE-QIDH+D3. b) PBE0+MBD-NL (non-local), PBE0+TS, PBE0+XDM (eXchange-hole Dipole Moment), ωB97X-D3, ωB97M-V, M06-2X, PBE+MBD. c) the semiempirical GFN2-xTB and Density Functional Tight Binding 3 (DFTB3)+MBD, and the classical force fields AMBER-GAFF2 and CHARMM-CGenFF.

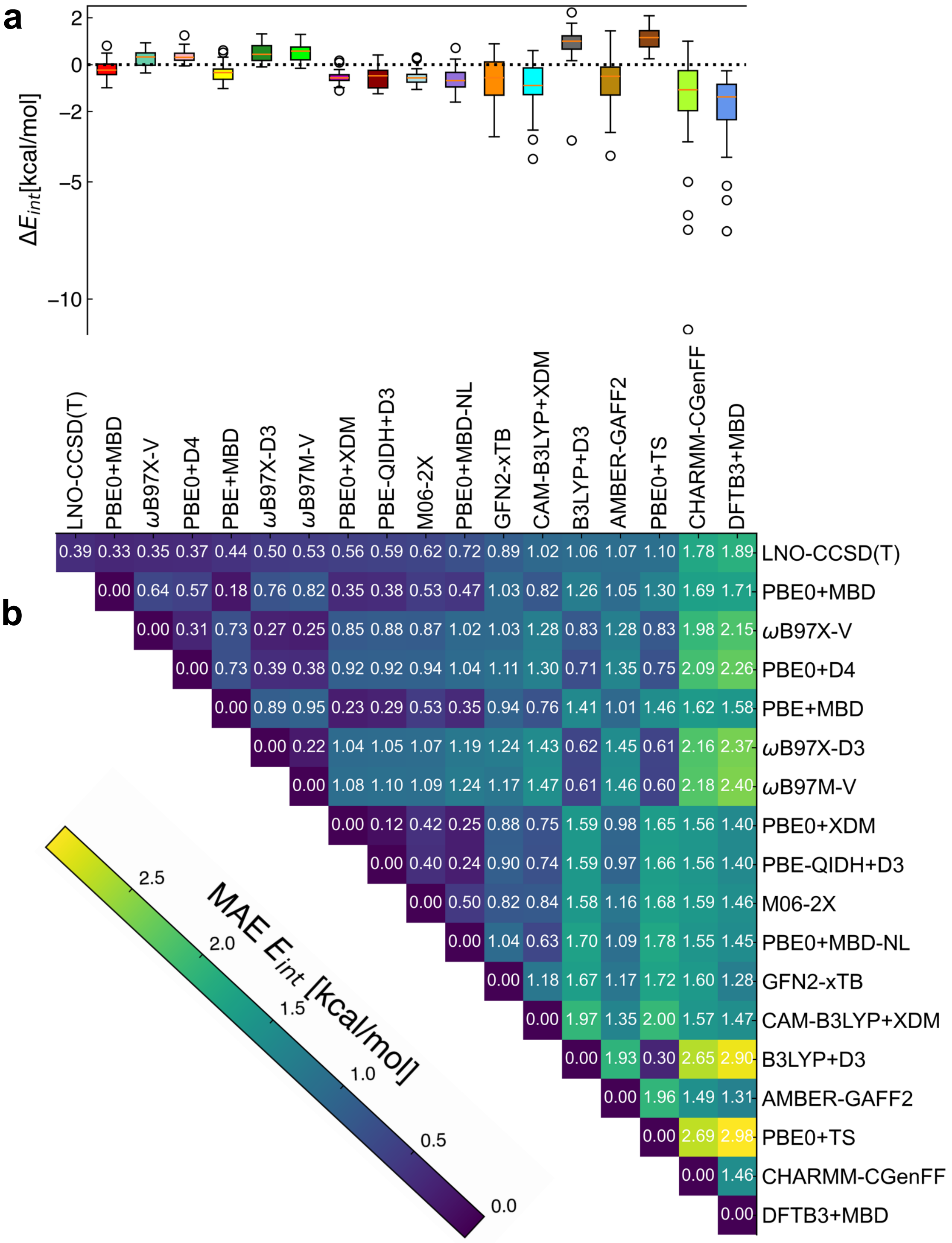

Figure S7. a) Distributions of interaction energy predictions w.r.t. Local Natural Orbitals - Coupled Cluster with Singles, Doubles, and perturbative triplets (LNO-CCSD(T)), ΔE_{int}, showed via box plots, for a selection of computational methods - Density Functional Theory (DFT) methods: PBE0+Many-Body Dispersion (MBD), ωB97X-V, PBE0+D4, PBE+MBD, ωB97X+D3, ωB97M-V, PBE0+eXchange-hole Dipole Moment (XDM), PBE-QIDH+D3, PBE0+MBD-NL(non-local), CAM-B3LYP+XDM, B3LYP+D3, PBE0+TS (Tkatchenko-Scheffler); semiempirical methods: Density Functional Tight Binding 3 (DFTB3)+MBD, GFN2-xTB; and classical force fields: AMBER-GAFF2 and CHARMM-CGenFF. The negative ΔE_{int} values signify underbinding, while the positive ones overbinding. b) A heatmap of Mean Absolute Error (MAE) values of predicted E_{int} w.r.t LNO-CCSD(T) for the 42 QUantum Interacting Dimer (QUID) equilibrium dimers in the first column, and the MAE of all methods w.r.t each other in subsequent columns. The computational methods to predict E_{int} were for the same methods as in a. *For the LNO-CCSD(T) method, the value shown with asterisk is the mean absolute of the uncertainty estimates for E_{int}.

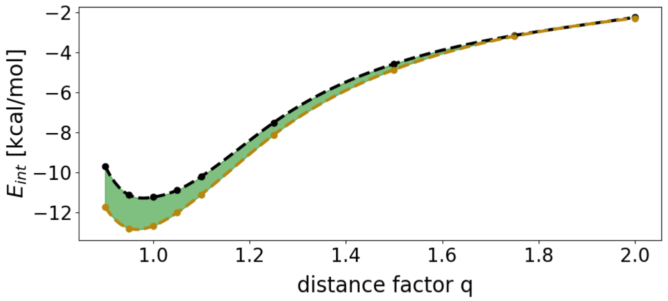

Figure S8. An example of the delta metric (as employed in literature: A. Khabibrakhmanov, et al. J. Chem. Theory Comput. 2023 19 (21), 7895-7907, DOI: 10.1021/acs.jctc.3c00797) for the F2B1 dimer, where the method shown in AMBER-GAFF2.

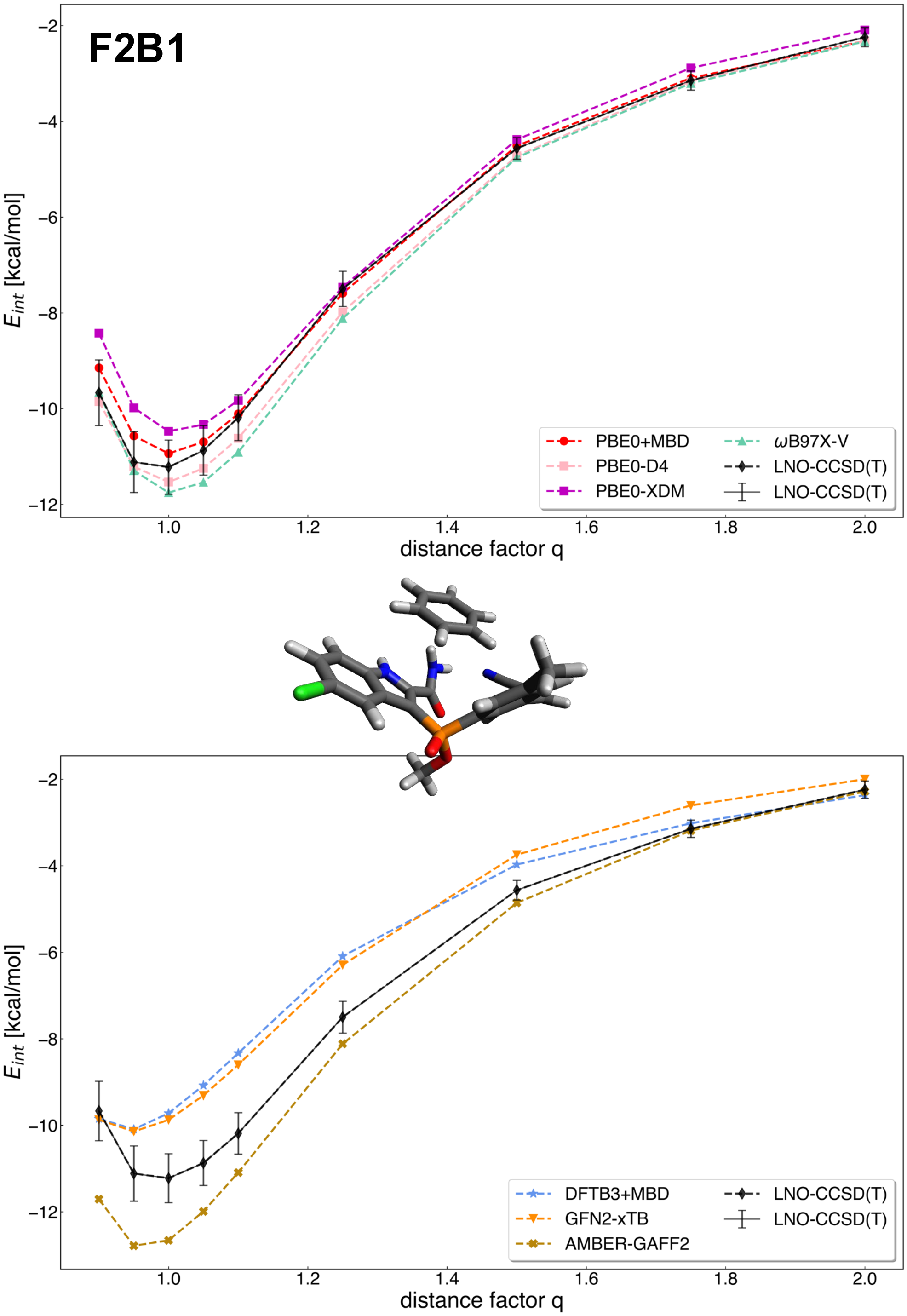

Figure S9. Dissociation curves for the the non-equilibrium dimer F2B1, for which Local Natural Orbitals - Coupled Cluster with Singles, Doubles, and perturbative triplets (LNO-CCSD(T)) level reference is shown. The Density Functional Theory (DFT) methods PBE0+Many-Body Dispersion (MBD), PBE0-D4, PBE0+eXchange-hole Dipole Moment (XDM), ωB97X-V in the top plot, and semi-empirical methods GFN2-xTB, and Density Functional Tight Binding 3 (DFTB3)+MBD, as well as the classical force field AMBER-GAFF2 in the bottom plot, are presented.

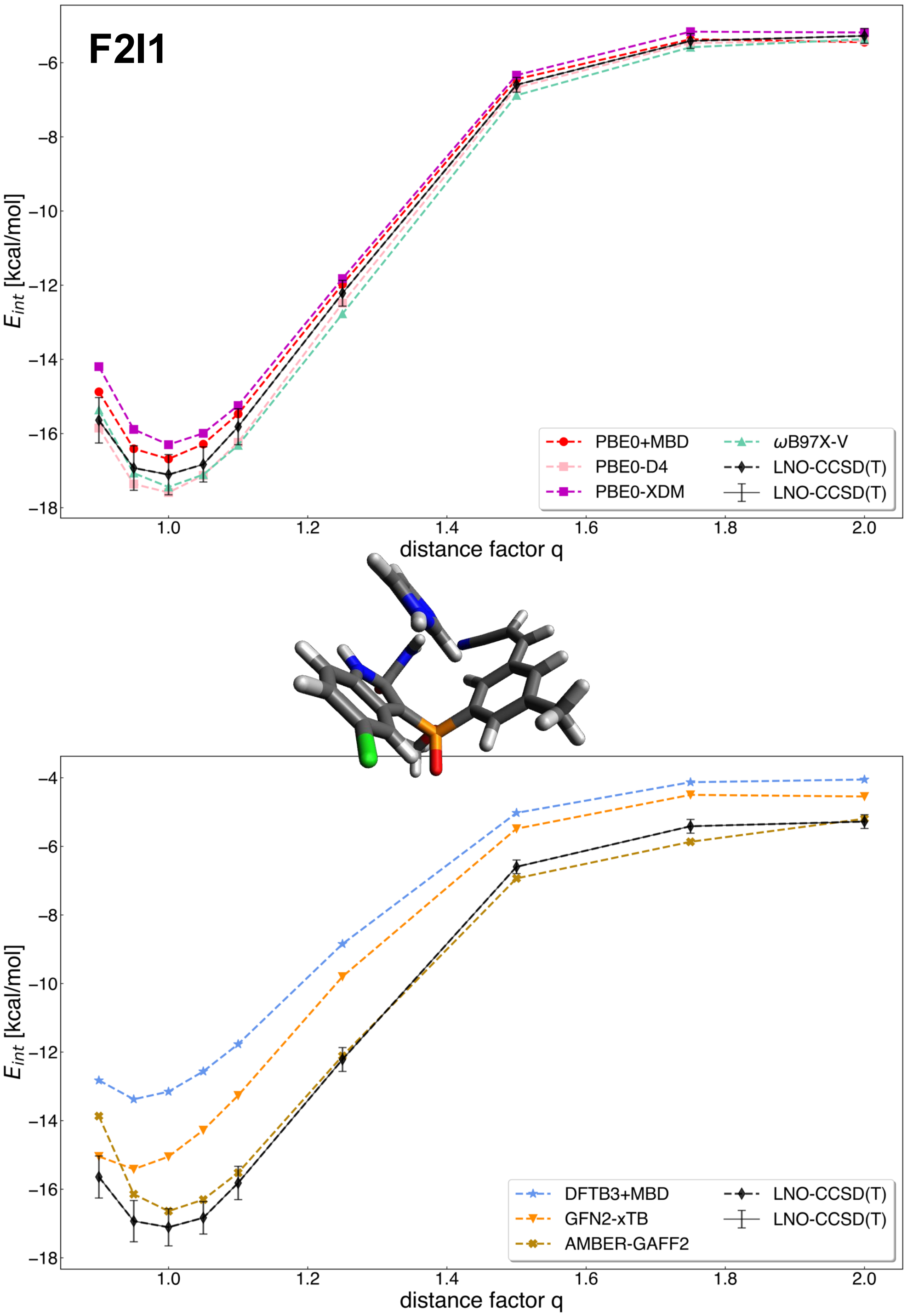

Figure S10. Dissociation curves for the the non-equilibrium dimer F2I1, for which Local Natural Orbitals - Coupled Cluster with Singles, Doubles, and perturbative triplets (LNO-CCSD(T)) level reference is shown. The DFT methods PBE0+Many-Body Dispersion (MBD), PBE0+D4, PBE0+eXchange-hole Dipole Moment (XDM), ωB97X-V in the top plot, and semi-empirical methods GFN2-xTB, and Density Functional Tight Binding 3 (DFTB3)+MBD, as well as the classical force field AMBER-GAFF2 in the bottom plot, are presented.

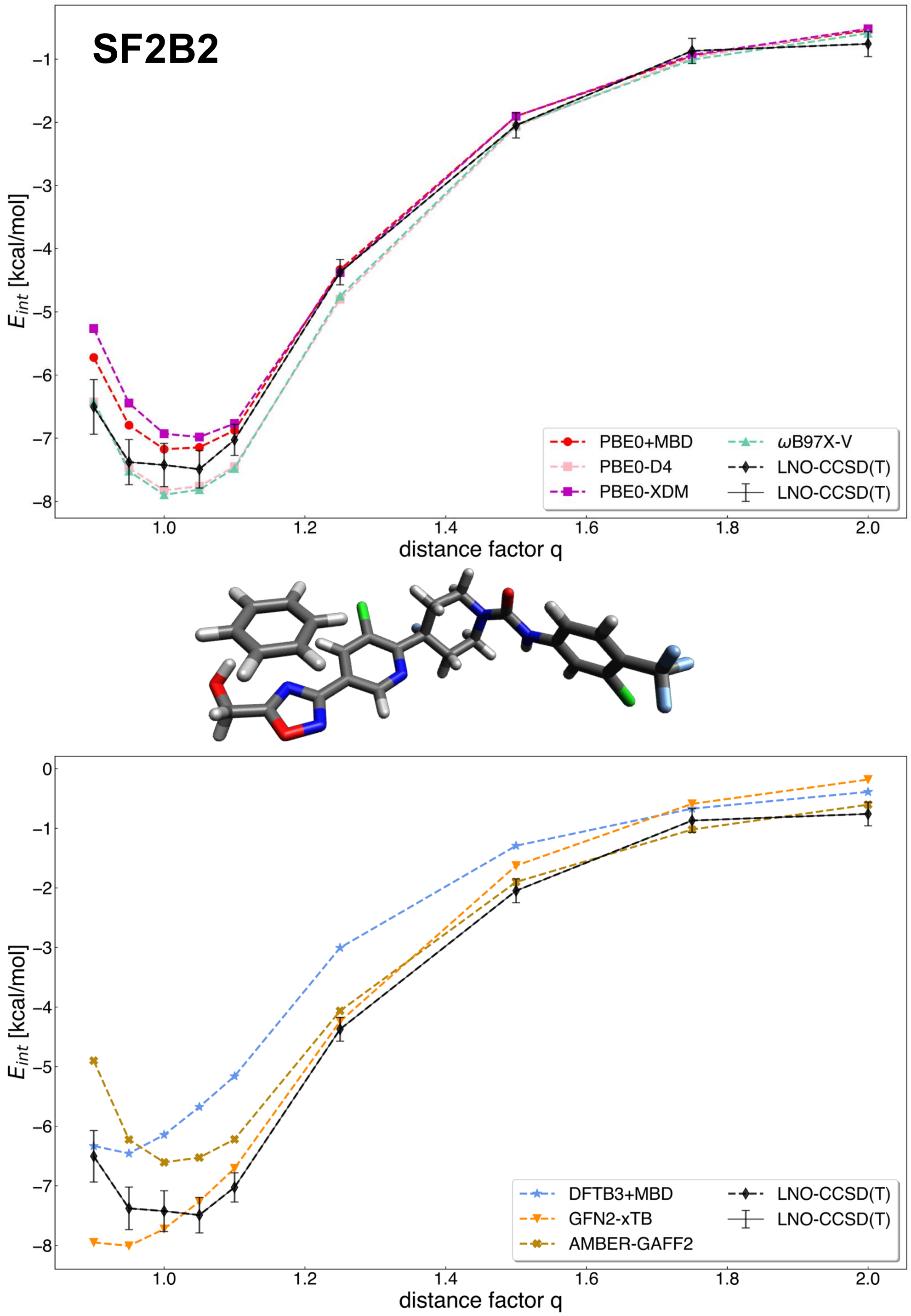

Figure S11. Dissociation curves for the the non-equilibrium dimer SF2B2, for which Local Natural Orbitals - Coupled Cluster with Singles, Doubles, and perturbative triplets (LNO-CCSD(T)) level reference is shown. The Density Functional Theory (DFT) methods PBE0+Many-Body Dispersion (MBD), PBE0+D4, PBE0+eXchange-hole Dipole Moment (XDM), ωB97X-V in the top plot, and semi-empirical methods GFN2-xTB, and Density Functional Tight Binding 3 (DFTB3)+MBD, as well as the classical force field AMBER-GAFF2 in the bottom plot, are presented.

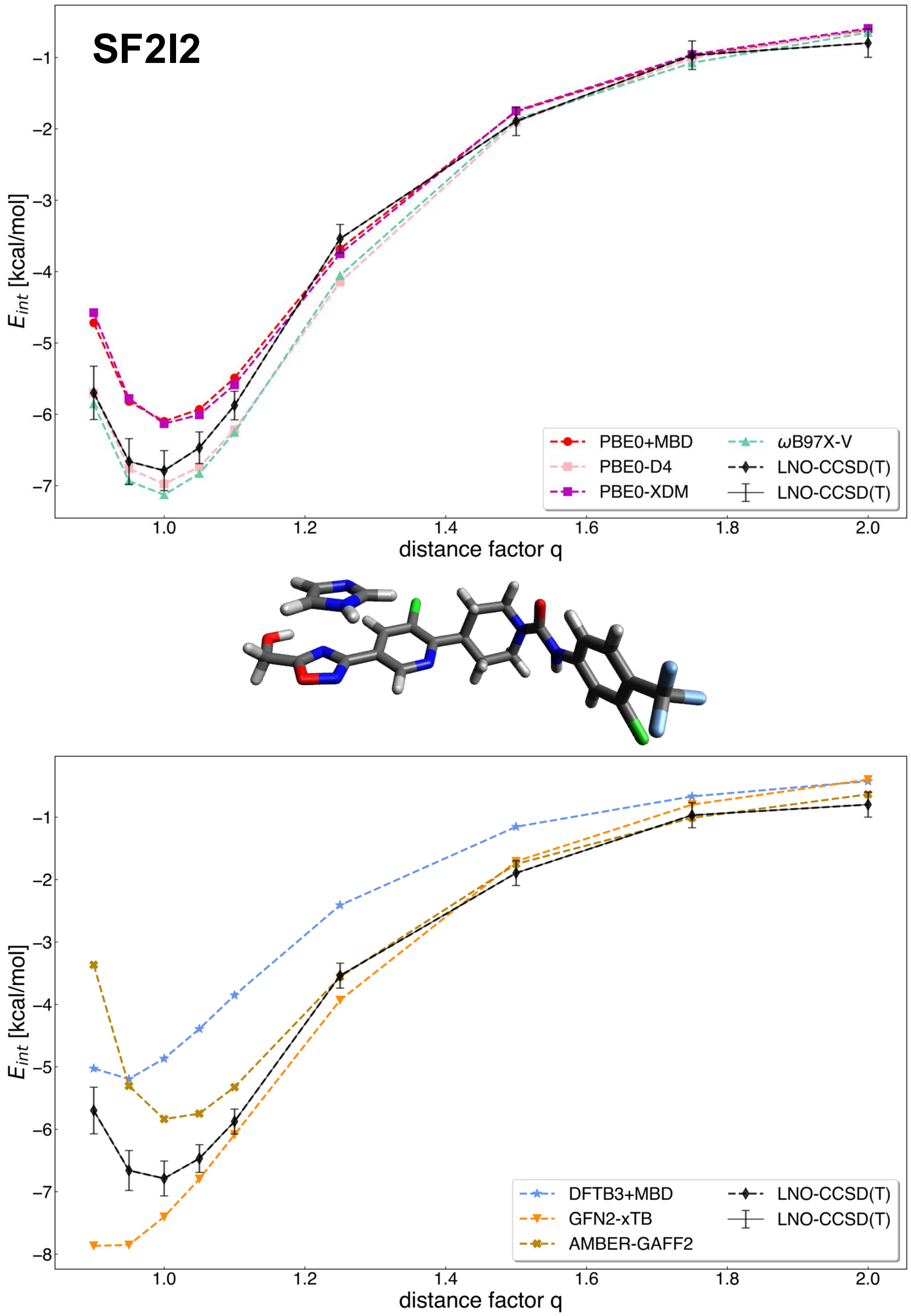

Figure S12. Dissociation curves for the the non-equilibrium dimer SF2I2, for which Local Natural Orbitals - Coupled Cluster with Singles, Doubles, and perturbative triplets (LNO-CCSD(T)) level reference is shown. The Density Functional Theory (DFT) methods PBE0+Many-Body Dispersion (MBD), PBE0+D4, PBE0+eXchange-hole Dipole Moment (XDM), ωB97X-V in the top plot, and semi-empirical methods GFN2-xTB, and Density Functional Tight Binding 3 (DFTB3)+MBD, as well as the classical force field AMBER-GAFF2 in the bottom plot, are presented.

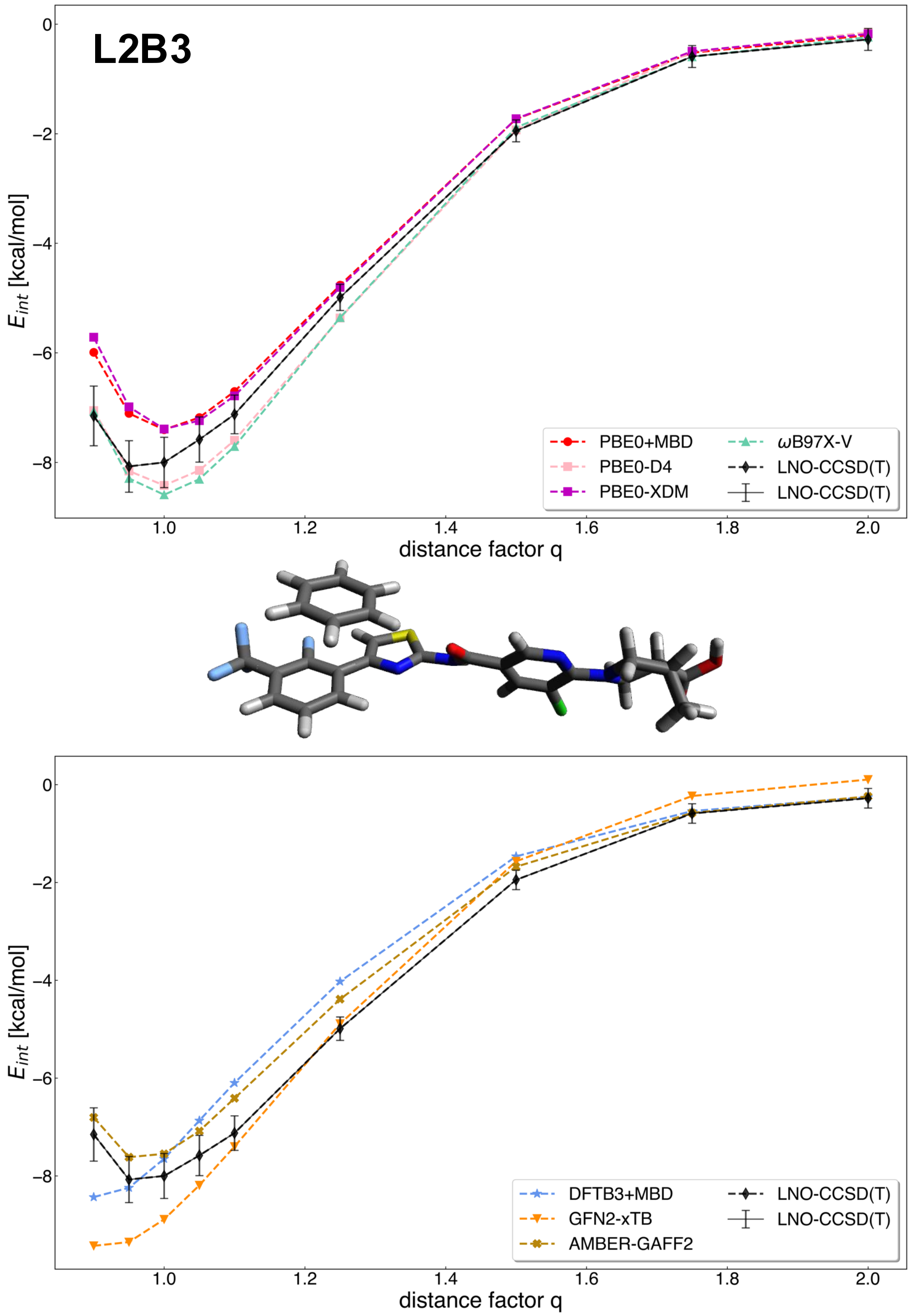

Figure S13. Dissociation curves for the the non-equilibrium dimer L2B3, for which Local Natural Orbitals - Coupled Cluster with Singles, Doubles, and perturbative triplets (LNO-CCSD(T)) level reference is shown. The Density Functional Theory (DFT) methods PBE0+Many-Body Dispersion (MBD), PBE0+D4, PBE0+eXchange-hole Dipole Moment (XDM), ωB97X-V in the top plot, and semi-empirical methods GFN2-xTB, and Density Functional Tight Binding 3 (DFTB3)+MBD, as well as the classical force field AMBER-GAFF2 in the bottom plot, are presented.

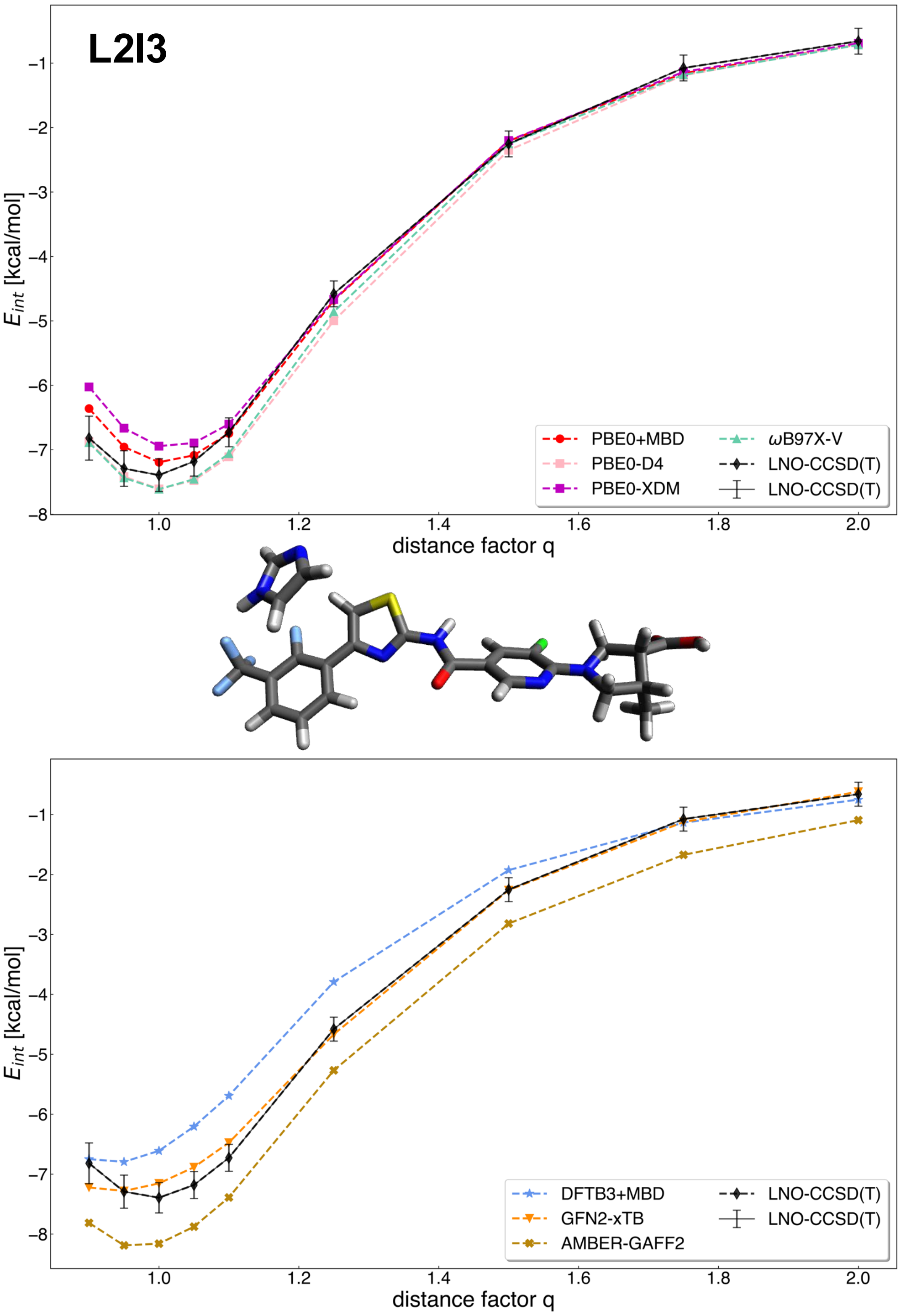

Figure S14. Dissociation curves for the the non-equilibrium dimer L2I3, for which Local Natural Orbitals - Coupled Cluster with Singles, Doubles, and perturbative triplets (LNO-CCSD(T)) level reference is shown. The Density Functional Theory (DFT) methods PBE0+Many-Body Dispersion (MBD), PBE0+D4, PBE0+eXchange-hole Dipole Moment (XDM), ωB97X-V in the top plot, and semi-empirical methods GFN2-xTB, and Density Functional Tight Binding 3 (DFTB3)+MBD, as well as the classical force field AMBER-GAFF2 in the bottom plot, are presented.

3 QUID property predictions

Among the equilibrium dimers, the highest α values are shown by the benzene-based linear conformations L2B1, L2B2, and L2B3 ($\alpha \sim 510\ a_0^3$). At first glance, this can be understood by their large radius of gyration R_g and the presence of more polarizable heavy atoms such as S and Cl. However, we have found that the semi-folded conformations SF1I1 and SF1I2, which are systems with intermediate R_g (see Fig.S4), display high dipole moments and low α values ($\sim 380\ a_0^3$), contradicting the physical intuition that systems with a more folded structure would have the lowest α values. Interestingly, SF1I1 presents a larger dipole moment than SF1I2, but the E_{int} of SF1I2 is more than two times the corresponding value of SF1I1 (see Fig. S18 of the SI)— an effect that may be a consequence of the highly electronegative groups at two ends, namely the trifluoromethyl $-CF_3$ and the sulfonyl $-SO_2$ groups. The smallest dipole moment is observed in F1I1, the only binding site on the F1 monomer that results in binding of both strong π–π stacking and 2 H-bonds. For the non-equilibrium selection configurations of depicted on Fig. S15d), F2I1, F2B1, SF2B2, SF2I2, L2B3, and L2I3, we can see the effect of the dissociation of the non-covalent bond on the molecular polarizability and dipole moment ranges.

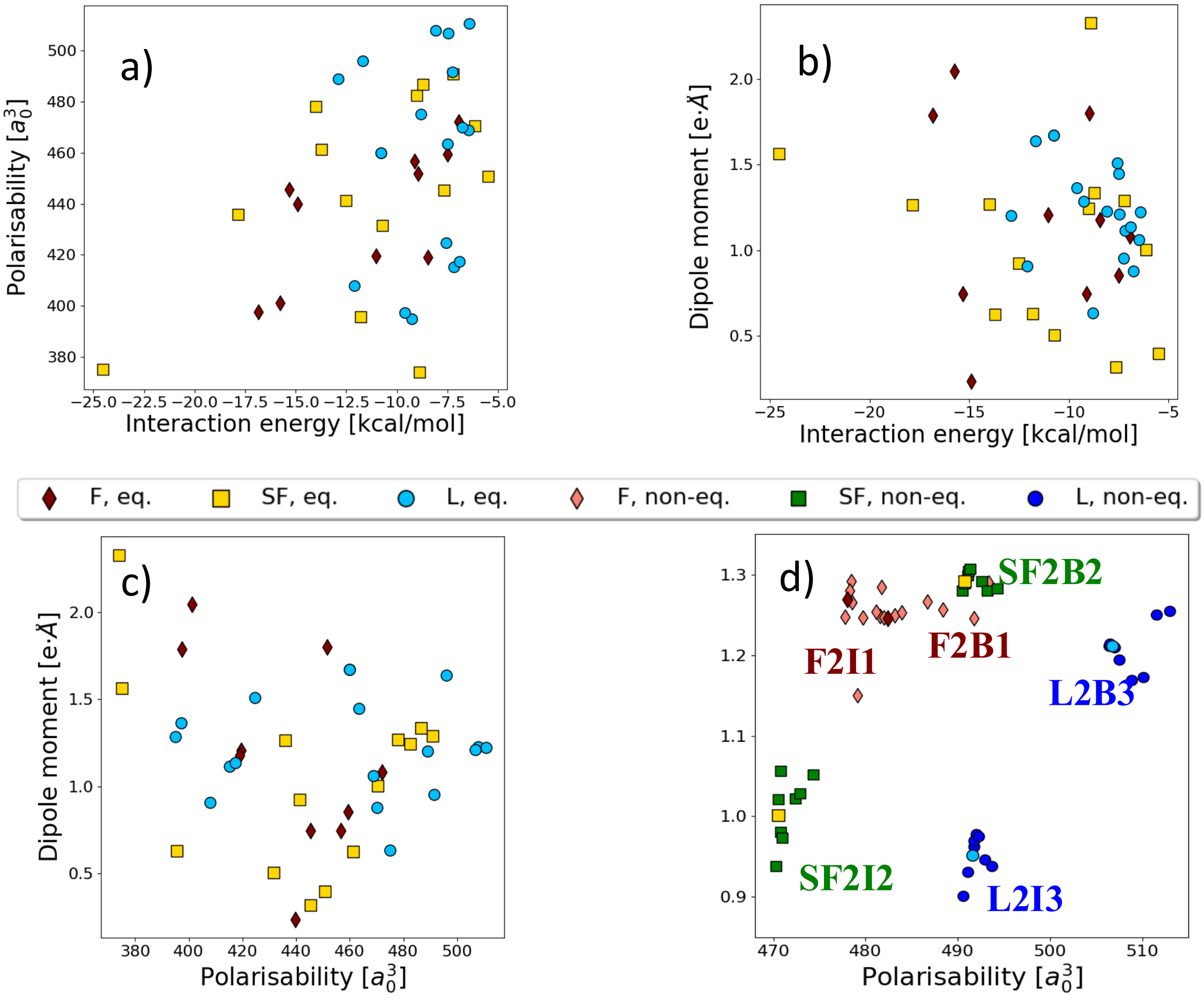

Figure S15. a) and b): Molecular properties correlation plot for all 42 QUantum Interacting Dimer (QUID) equilibrium dimers colour-coded by geometry category for a)total dipole moment versus interaction energy and b) molecular polarizability versus interaction energy. c) and d): Molecular properties correlation plot of the total dipole moment versus molecular polarizability for a) all equilibrium QUID dimers, folded configurations in red, semi-folded in yellow, and linear in blue; and b) for the equilibrium and non-equilibrium configurations of six configurations F2B1 and F2I1 in pink, SF2B1 and SF2I1 in green, L2B3 and L2I3 in dark blue.

#	Symbol	Property	Unit	Level	HDF5 keys
1	E_{int}	Interaction energy	eV	PBE0+MBD	'PBE0+MBD'
2	E_{int}	Interaction energy	eV	PBE0	'PBE0'
3	E_{int}	Interaction energy	eV	PBE0+MBD with BSSE	'PBE0+BSSE'
4	E_{int}	Interaction energy	eV	PBE0+MBD-NL	'PBE0+MBD_NL'
5	E_{int}	Interaction energy	eV	PBE0+D4	'PBE0+D4'
6	E_{int}	Interaction energy	eV	PBE0+XDM	'PBE0+XDM'
7	E_{int}	Interaction energy	eV	M06-2X	'M06_2X'
8	E_{int}	Interaction energy	eV	ωB97X-V	'wB97X_V'
9	E_{int}	Interaction energy	eV	ωB97M-V	'wB97M_V'
10	E_{int}	Interaction energy	eV	ωB97X-D3	'wB97X_D3'
11	E_{int}	Interaction energy	eV	PBE+MBD	'PBE+MBD'
12	E_{int}	Interaction energy	eV	PBE-QIDH+D3	'PBE_QIDH+D3'
13	E_{int}	Interaction energy	eV	B3LYP+D3	'B3LYP+D3'
14	E_{int}	Interaction energy	eV	CAM-B3LYP+XDM	'CAM_B3LYP+XDM'
15	E_{int}	Interaction energy	eV	BHandHLYP+XDM	'BHandHLYP+XDM'
16	E_{int}	Interaction energy	eV	PBE0+TS	'PBE0+TS'
17	E_{int}	Interaction energy	eV	Hartree-Fock	'HF'
18	E_{int}	Interaction energy	eV	DFTB3	'DFTB3'
19	E_{int}	Interaction energy	eV	DFTB3+MBD	'DFTB3+MBD'
20	E_{int}	Interaction energy	eV	GFN2-xTB	'GFN2_xTB'
21	E_{int}	Interaction energy	eV	sSAPT0	'SAPT'
22	E_{int}	Interaction energy	eV	LNO-CCSD	'CCSD'
23	E_{int}	Interaction energy	eV	LNO-CCSD(T)/CBS	'CCSDT'
24	E_{int}	Interaction energy	eV	ΔLNO-CCSD(T)/CBS	'deltaCCSDT'
25	E_{int}	Interaction energy	eV	FN-DMC-0.025$\delta\tau$	'DMC'
26	E_{int}	Interaction energy	eV	δFN-DMC$\delta\tau$	'deltaDMC'
27	E_{int}	Interaction energy	eV	AMBER-GAFF2	'GAFF2'
28	E_{int}	Interaction energy	eV	CHARMM-CGenFF	'CHARMM_CGenFF'

Table S4. List of interaction energy levels in the QUantum Interacting Dimer (QUID) dataset. Each interaction energy E_{int} is represented by a symbol (with units and dimension) and can be found in the HDF5 QUID file using the corresponding HDF5 keys under the 'Eint' key. The Density Functional Theory (DFT) calculations were carried out with `FHI-aims` on tight settings or `Psi4` with quadruple-zeta def2-QZVPPD basis set.

#	Symbol	Property	Unit	Dimension	Type	Level	HDF5 keys
1	Z	Atomic numbers	-	N	S	-	'atNUM'
2	R	Atomic positions (coordinates)	Å	$3N$	S	TB	'atXYZ'
5	E_{tot}	Total PBE0+MBD energy	eV	1	M,G	P0M	'ePBE0+MBD'
6	E_{TB}	Total DFTB3+MBD energy	eV	1	M,G	TB	'eDFTB+MBD'
7	E_{at}	Atomization energy	eV	1	M,G	P0	'eAT'
8	E_{PBE0}	PBE0 energy	eV	1	M,G	P0	'ePBE0'
9	E_{MBD}	MBD energy	eV	1	M,G	P0M	'eMBD'
10	E_{TS}	TS dispersion energy	eV	1	M,G	P0	'eTS'
11	E_{nn}	Nuclear-nuclear repulsion energy	eV	1	M,G	-	'eNN'
12	E_{kin}	Kinetic energy	eV	1	M,G	P0	'eKIN'
13	E_{ne}	Nuclear-electron attraction	eV	1	M,G	P0	'eNE'
14	E_{coul}	Classical coulomb energy (el-el)	eV	1	M,G	P0	'eEE'
15	E_{xc}	Exchange-correlation energy	eV	1	M,G	P0	'eXC'
16	E_{x}	Exchange energy	eV	1	M,G	P0	'eX'
17	E_{c}	Correlation energy	eV	1	M,G	P0	'eC'
18	E_{xx}	Exact exchange energy	eV	1	M,G	P0	'eXX'
19	E_{KS}	Sum of Kohn-Sham eigenvalues	eV	1	M,G	P0	'eKSE'
20	ε	Kohn-Sham eigenvalues	eV	*	M,G	P0	'KSE'
21	E_{HOMO}	HOMO energy	eV	1	M,G	P0	'eH'
22	E_{LUMO}	LUMO energy	eV	1	M,G	P0	'eL'
23	E_{gap}	HOMO-LUMO gap	eV	1	M,G	P0	'HLgap'
24	D_{s}	Scalar dipole moment	$e \cdot$ Å	1	M,G	P0	'DIP'
25	D	Dipole moment	$e \cdot$ Å	3	M,G	P0	'vDIP'
26	Q_{tot}	Total quadrupole moment	$e \cdot$ Å^2	3	M,G	P0	'vTQ'
27	Q_{ion}	Ionic quadrupole moment	$e \cdot$ Å^2	3	M,G	P0	'vIQ'
28	Q_{elec}	Electronic quadrupole moment	$e \cdot$ Å^2	3	M,G	P0	'vEQ'
29	C_6	Molecular C_6 coefficient	$E_h \cdot a_0^3$	1	M,R	P0M	'mC6'
30	α_{s}	Molecular polarizability (isotropic)	a_0^3	1	M,R	P0M	'mPOL'
31	α	Molecular polarizability tensor	a_0^3	9	M,R	P0M	'mTPOL'
32	F_{tot}	Total PBE0+MBD atomic forces	eV/Å	$3N$	A,G	P0M	'totFOR'
33	F_{PBE0}	PBE0 atomic forces	eV/Å	$3N$	A,G	P0	'pbe0FOR'
34	F_{MBD}	MBD atomic forces	eV/Å	$3N$	A,G	P0M	'FvdWMBD'
35	F_{D4}	D4 atomic forces	eV/Å	$3N$	A,G	P0D4	'FvdWD4'
36	F_{XDM}	XDM atomic forces	eV/Å	$3N$	A,G	P0XDM	'FvdWXDM'
37	V_{H}	Hirshfeld volumes	a_0^3	N	A,G	P0	'hVOL'
38	V_{ratio}	Hirshfeld ratios	-	N	A,G	P0	'hRAT'
39	q_{H}	Hirshfeld charges	e	N	A,G	P0	'hCHG'
40	$D_{\text{H,s}}$	Scalar Hirshfeld dipole moments	$e \cdot a_0$	N	A,G	P0	'hDIP'
41	D_{H}	Hirshfeld dipole moments	$e \cdot a_0$	$3N$	A,G	P0	'hVDIP'
42	$\widetilde{C_6}$	Atomic C_6 coefficients	$E_h \cdot a_0^6$	N	A,R	P0M	'atC6'
43	$\widetilde{\alpha}_{\text{s}}$	Atomic polarizabilities (isotropic)	a_0^3	N	A,R	P0M	'atPOL'
44	R_{vdW}	vdW radii	a_0	N	A,R	P0M	'vdwR'

*The number of Kohn-Sham eigenvalues varies for each molecule.

Table S5. List of physicochemical properties in the QUID dataset. Each property is represented by a symbol (with units and dimension) and can be found in the HDF5 QUID file using the corresponding HDF5 keys under the 'properties' key. Different property types are distinguished as follows: structural (S), molecular (M), atom-in-a-molecule (A), ground-state (G), and response (R). Different levels of theory are indicated as follows: DFTB3+MBD (TB), PBE0 (P0), PBE0+MBD (P0M), PBE0+D4 (P0D4), and PBE0+XDM (P0XDM). The P0M label indicates which properties explicitly include dispersion interactions. P0D4 and P0XDM are only used to indicate the vdW force components at the corresponding level. E_h and a_0 refer to the atomic units of energy (Hartree) and length (Bohr radius), respectively.

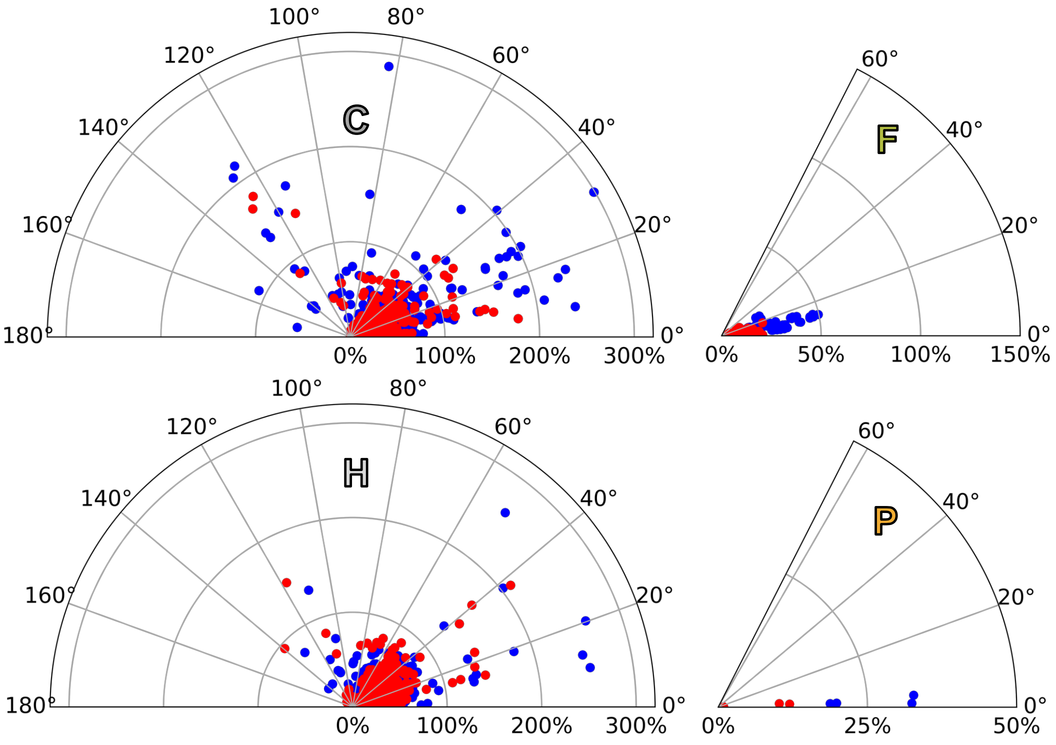

Figure S16. Atomic forces radial plot of the differences between the forces of PBE0+D4 (blue) and PBE0+XDM (eXchange-hole Dipole Moment (XDM)) (red) w.r.t PBE0+MBD plotted as an arccos of the forces vectors versus a radius of the differences of the magnitudes scaled by the PBE0+MBD (Many-Body Dispersion) magnitude and provided in percentages. The plots are for the atomic forces for H, C, F, and P atoms in all equilibrium molecules.

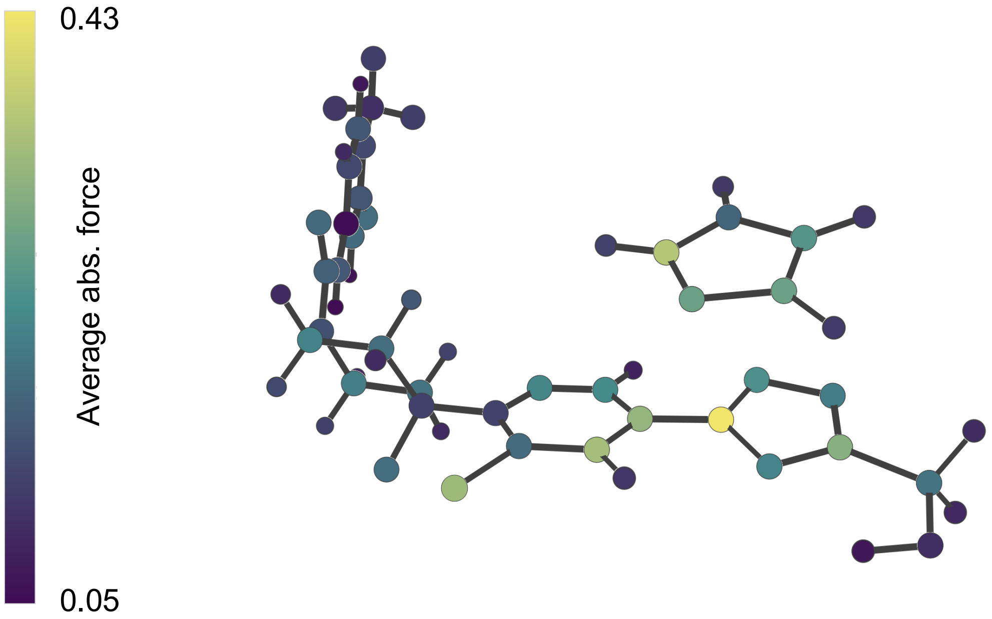

Figure S17. An image of the force vectors corresponding to the MBD (Many-Body Dispersion) force for the SF2I2 dimer, produced with `FFAST` software.

4 Supporting information for Section "Computational Methods"

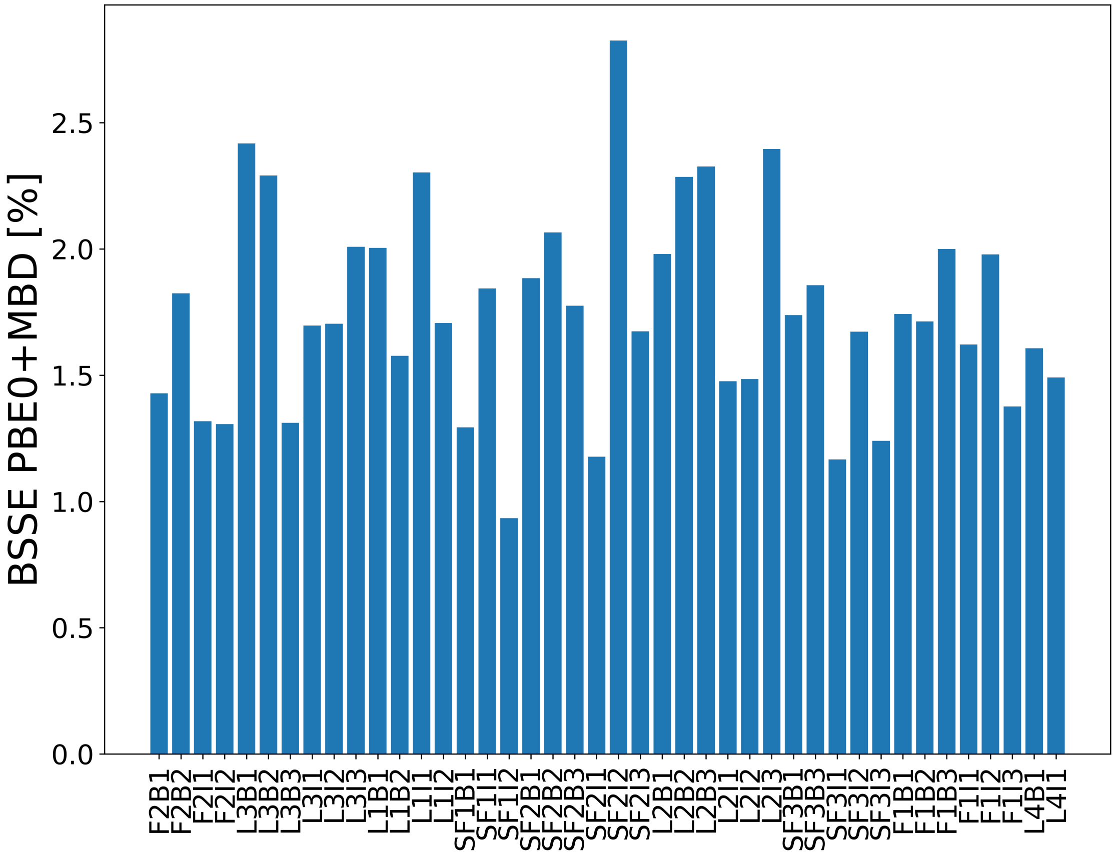

Figure S18. BSSE, Basis set superposition error, (also known as counterpoise correction) for the interaction energies at PBE0+Many-Body Dispersion (MBD) for all 42 equilibrium QUantum Interacting Dimer (QUID) structures, shown in % by dimer.

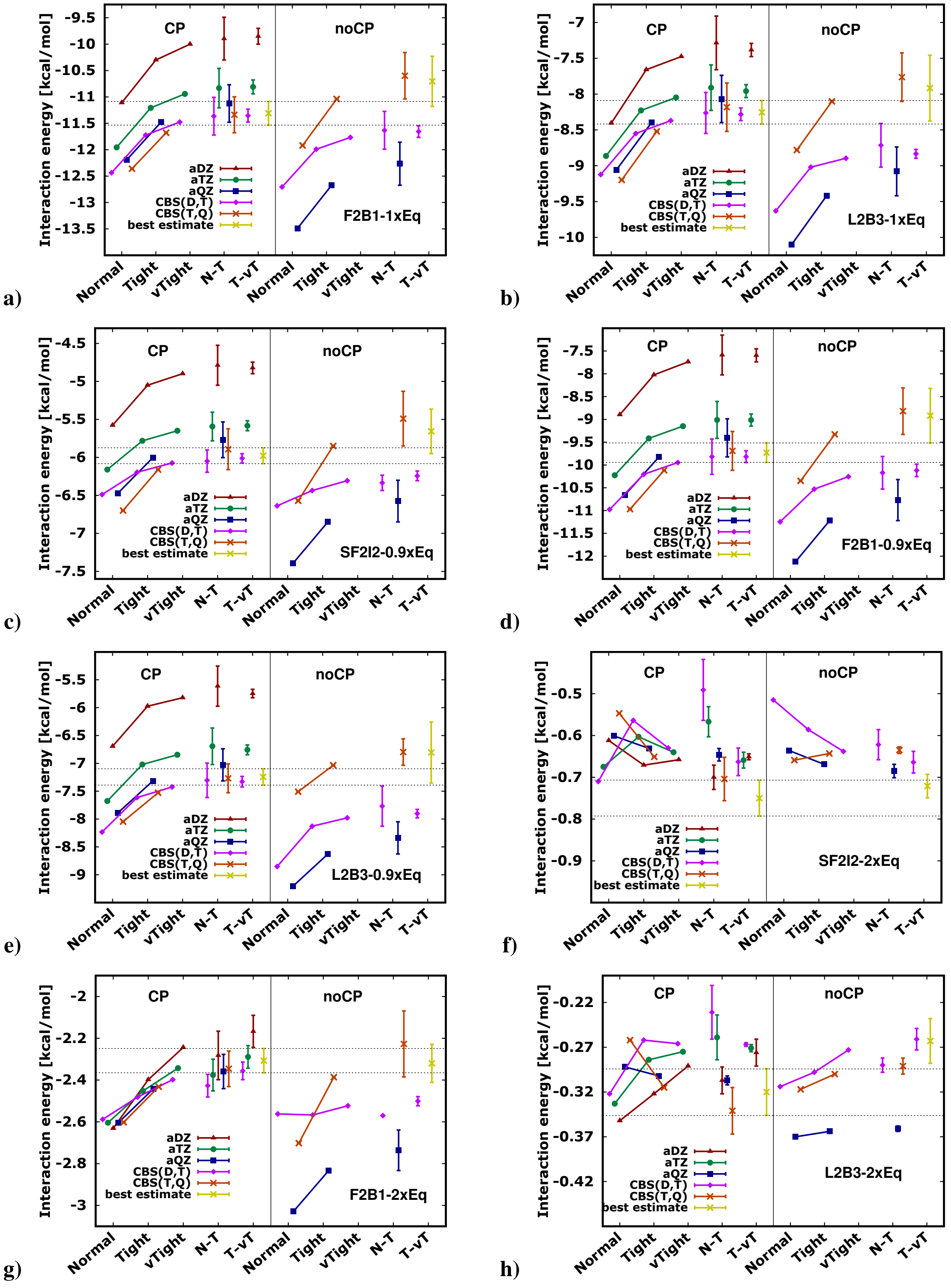

Figure S19. Local Natural Orbitals - Coupled Cluster with Singles, Doubles, and perturbative triplets (LNO-CCSD(T)) interaction energy convergence analysis with respect to the LNO thresholds (x axis) and aug-cc-pV(X+d)Z (aXZ) basis set choices with (CP, left) and without (noCP, right) counterpoise corrections, including the best estimate interaction energy [Eq. (2) of the main text]. In separate panels are presented the dimers F2B1 in panel a), L2B3 in panel b), and SF2I2 at equilibrium distance is given in Fig.7 of the main text for 1× the equilibrium geometry intermonomer distance; SF2I2 in panel c), F2B1 in panel d), and L2B3 in panel e) for 0.9× the equilibrium geometry intermonomer distance; and SF2I2 in panel f), F2B1 in panel g), and L2B3 in panel h) for 2.0× the equilibrium geometry intermonomer distance.
